# The value of mesenteric closure after laparoscopic right hemicolectomy: a scoping review

**DOI:** 10.1186/s12893-023-02033-3

**Published:** 2023-05-17

**Authors:** Weimin Xu, Jianping Zhou

**Affiliations:** 1 Department of Gastrointestinal Surgery, The First Hospital, China Medical University, Shenyang, Liaoning Province China; 2 Department of Health Statistics, School of Public Health, China Medical University, Shenyang, China

**Keywords:** Laparoscopic right hemicolectomy, Mesenteric defects, Mesenteric closure, Scoping review, Prognostic impact

## Abstract

**Objective:**

To evaluate the prognostic impact and describe suturing tools of mesenteric closure after laparoscopic right hemicolectomy (LRH).

**Methods:**

PubMed, Embase, Cochrane library, Web of Science, and Scopus databases, were searched and publications relating to mesenteric closure data and tools were extracted. Search terms: “Mesenteric Defects” and “Mesenteric Closure” were used, and manual searches of eligible articles from literature reference lists performed.

**Result:**

A total of 7 publications were identified. 5 focused on prognostic impact and 4 referred to tools for mesenteric closure, two of which concerned both prognostic data and tools. All studies related to prognostic impact were single center with “low” modified GRADE quality. A high degree of heterogeneous was found.

**Conclusion:**

The evidence from current research does not support routine closure of mesenteric defects. Use of a polymer ligation clip has produced favorable results in a small sample size trial and further investigation is merited. A large randomized controlled trial is still warranted.

## Introduction

Laparoscopy is commonly used in colorectal surgery, having advantages over open surgery [1, 2]. Mesenteric closure during laparoscopic colorectal surgery may prevent the occurrence of internal hernia caused by the small intestine passing through the mesenteric defect [3]. However, closure is challenging. When a gap of 2–5 cm occurs due to an incomplete procedure, the risk of internal hernia will be increased [4]. Thus, debate persists as to whether mesenteric defects should be closed during laparoscopic colorectal surgery. Mesenteric anatomical structure may differ in individual cases of intestinal resection, prompting some clinicians to maintain that evaluations should be performed on a case by case basis for different types of laparoscopic colorectal surgery [5]. “Omentum majus filling” has been suggested for laparoscopic transverse colectomy [6], and laparoscopic anterior rectal resection proposed “mesenteric closure routinely” [7]. A case-series analysis of laparoscopic left hemicolectomy has been conducted [8], but studies of laparoscopic right hemicolectomy (LRH) are limited and the merits of mesenteric closure from an evidence-based perspective deserve scrutiny. The current review includes a systematic analysis of prognostic data relating to mesenteric defects in LRH. Past approaches are discussed and future perspectives analyzed.

## Methodology

The current scoping review was conducted according to some articles and PRISMA-ScR guidelines [9–11]. The prospective nature of a scoping review does not encompass literature quality scoring [12] and all studies meeting inclusion criteria were summarized and discussed. The review focused on (1) prognostic impact of mesenteric closure/mesenteric defects of patients after LRH, and (2) tools for mesenteric closure.

### Search strategy

PubMed, Embase, Cochrane library, Web of Science and Scopus databases were searched using the search terms: “Mesenteric Defects” and “Mesenteric Closure” connected by “OR”. English language publications without limit of time were specified with the scope of title, keyword and abstract. Eligible publications were read in full and further studies identified from the reference lists. The search was conducted in October 2022.

### Inclusion and exclusion criteria

Inclusion criteria were as follows: (1) data relating to mesenteric defects or information regarding tools for mesenteric closure in LRH; (2) publications containing prognostic data on LRH; (3) publications other than secondary literature or case reports; (4) English language.

### Data extraction and analysis

Data extraction charts were developed by the authors and disagreements resolved by discussion. A high degree of clinical heterogeneity was apparent, therefore, data was not merged into a meta-analysis but summarized by forest plot. Data extraction was performed by Microsoft Excel 2010 software. Categorical binary data was compared with Fisher’s exact test or χ2 test as appropriate. R (version:4.2.0, package: forestplot) was used to construct the forest plot and perform statistical analysis.

## Results

A total of 3587 citations were initially retrieved during October 2022 with an additional 2 added from reference lists. Duplicates were removed and 7 citations met the inclusion criteria, of which 5 contained relevant data, and 4 referred to tools related to mesenteric closure on LRH. Two publications referred to both prognostic data and tools.

**Fig. 1:**
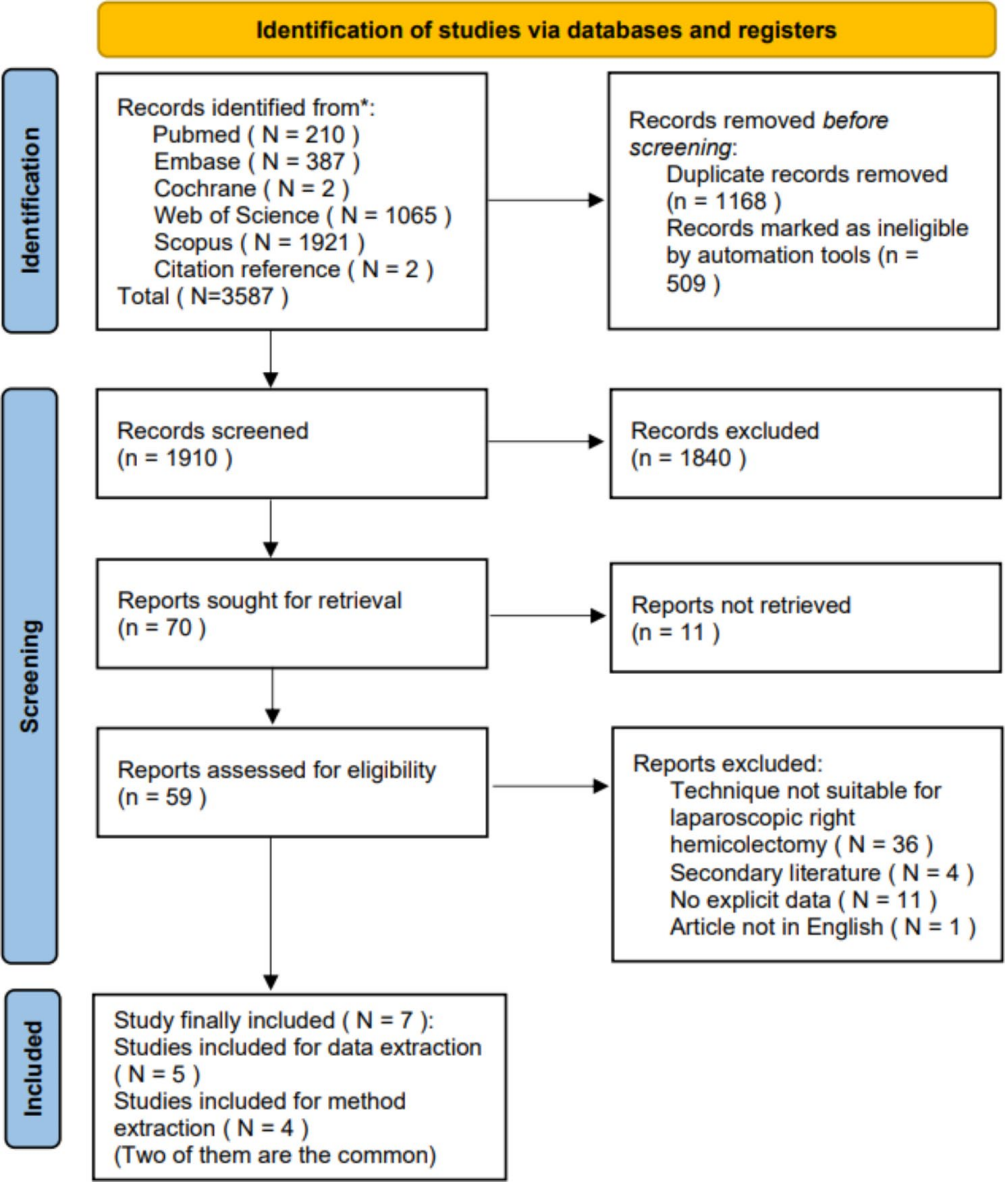
PRISMA2020 Flow diagram showing publication selection

### Characteristics of eligible studies

Publications from the time period 2009 to 2021 were included, of which 57% were published in the five years from 2016–2021 [13–16]. Three studies from North America [5, 8, 13], one from Asia [15] and three from Europe were finally selected [14, 16, 17]. All studies used for data extraction were retrospective with “low” quality judged by Modified GRADE quality assessment [18].

### Complications after mesenteric closure in LRH

Details of patients who underwent mesenteric closure have been collated in Table 1. Only those cases for whom clear details of mesenteric defects were given have been included. There is some diversity in the complications listed arising from different research objectives and intervention measures in the literature. Specific complications have been listed and reviewed in detail in addition to those relating to the mesenteric defects (Table 1).

**Table 1:** Post-operative complications in mesenteric closure patients

Author	Year	Study type	Sample size	Post-operative complications	Complications due to mesenteric defects	Reoperation	Death due to complications	Post-operative time of onset of complications	Rate of for the post operation complications
Sica et al. [14]	2021	Prospective	41	1x Bleeding; 3x Anastomotic leakage; 1x Abdominal abscess leakage Total:5	0	2x Anastomotic leakage; Total: 2	0	In one week	0.122
Sugiyama et al. [15]	2016	Retrospective	10	0	0	0	0	-	0
Vignali et al. [16]	2017	Retrospective	64	3x Anastomotic leakage; 4x Intestinal obstruction; 1x Hemorrhage; 6x Intra-abdominal infection; 5x Infection of surgical scar Total: 19	Not mentioned	0	0	In one month	0.297
Fabozzi et al. [17]	2010	Retrospective	50	0	0	0	0	-	0

Table 1 summarizes the data of 175 patients with mesenteric closure from four studies The incidence of postoperative complications varied from 0 to 30% and differences in the range of outcome records and small sample sizes may account for disparities. The difference recorded for the last two studies is quite large, and we suspected that incision infection had not been recorded in Sica et al.’s study [14]. The incidence of post-operative anastomotic leakage was 5% for both studies [14, 16]. Only one study gave a 5% reoperation rate [14] and there were no postoperative deaths, no internal hernias and no mention of complications related to the closure of the mesenteric defects. Serious complications were very rare and deaths did not occur in patients receiving mesenteric closure. However, the small sample size makes it hard to rule out the possibility of sampling errors.

### Complications arising from retention of mesenteric defects in LRH

The purpose of the current study was to explore LRH prognosis with mesenteric closure but data relating to postoperative complications in the absence of mesenteric closure are also relevant to this objective and are summarized in (Table 2).

**Table 2:** Post-operative complications arising from mesenteric defect patients

Author	Sugiyama et al. [15]	Vignali et al. [16]	Fabozzi et al. [17]	Cabot et al. [5]
Year	2016	2017	2010	2010
Study type	Retrospective	Retrospective	Retrospective	Retrospective
Sample size	9	64	50	530
Post-operative complications	1x ileus, 1x surgical site infection (SSI) Total: ≥2	5x anastomotic leakage; 2x Intestinal obstruction; 1x Hemorrhage; 11x Infection of surgical scar Total: 21	3x respiratory infection, 3x anastomotic leakage, 2x intestinal hernia, 3x mini-laparotomy infections, 1x postoperative femoral neurosis, 1x postoperative heart attack, 1x postoperative pancreatitis Total: 14	26x small bowel obstruction (SBO), 8x anastomotic leak, 6x myocardial infarction Total: 40
Complications due to mesenteric defects	1x ileus Total: 1	not mentioned	2x intestinal hernia Total: 2	2x SBO, 2x anastomosis torsion Total: 4
Reoperation	1x ileus, 1x SSI Total: 2	4x Anastomotic leakage Total: 4	3 (none clearly details) Total: 3	14x SBO Total:14
Death	0	0	0	1x multisystem organ failure Total: 1
Post-operative time of onset of complication	In one month	Mostly in one month	not mentioned	12x in one month; 21x in one year (the longest in 53 months)
Rate of post-operative complications	≥ 0.222^*†*^	≥ 0.328^*‡*^	0.280	0.075

*†* Only the reoperation was fully described

*‡* Complications occurring after one month were not fully described

Postoperative complication rates arising in patients who did not undergo mesenteric closure were 22 − 32% in three small sample size studies [15–17], Low complication rates among LRH patients with mesenteric defects described in the large case series study may be due to the limitations of the database used in which only the more serious complications were recorded [5]. However, restricting scrutiny to the incidence of postoperative intestinal obstruction or complications caused by mesenteric defects still gives great variability of values, making it difficult to derive a stable estimate of postoperative complications in the non-closure group. Only one postoperative death, due to multiple organ failure, was recorded among 653 patients [5]. Patients retaining mesenteric defects did not appear to experience a significant disadvantage compared with those receiving closure. However, every study involving LRH and retention of mesenteric defects has included cases where reoperation was necessary. Thus, retention ofmesenteric defects seems to lead to a higher reoperation rate judging by initial impressions.

### Comparison of prognosis of LRH

**Fig. 2:**
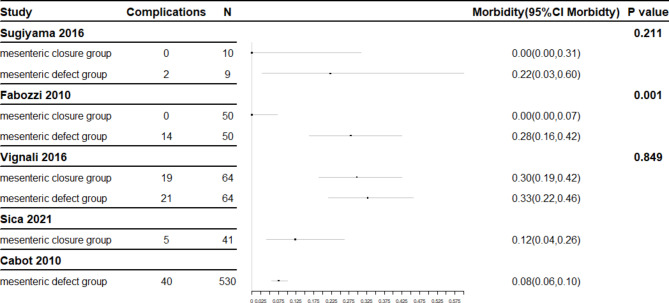
Forest plot of complications

**Fig. 3:**
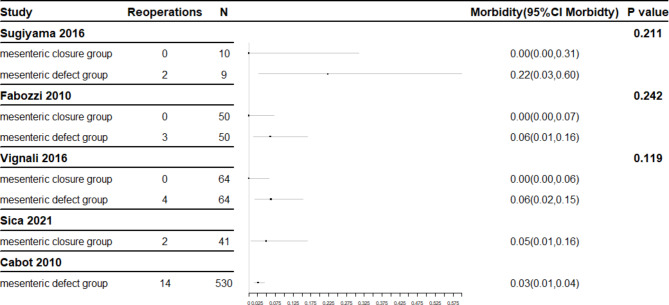
Forest plot of reoperations

Postoperative complication and postoperative reoperation incidences were compared. Comparisons in the three double-arm studies involving both closure and non-closure patients are possible but there are some limitations. Too few cases of LRH were included in the Sugiyama study [15]. Total laparoscopic surgery was performed for closure patients while laparoscopic-assisted right hemicolectomy was performed for non-closure patients in the Fabozzi study [17]. The Vignali study [16] only included patients with BMI > 30 with the confounder that some patients accepted intra-anastomosis and others extra-anastomosis. The large degree of clinical heterogeneity makes merging the data into a meta-analysis unsuitable and comparisons may only be made within a single study. Postoperative complications and postoperative reoperation rates are shown in the forest plots represented in Figs. 2 and 3.

Only one study showed a significant statistical difference in postoperative complications (Fig. 1). A lower rate of postoperative complications was found for patients receiving closure but there were many confounding factors and this was a single-institution study [14]. Thus, the evidence-based demonstration level was not high enough to be convincing. Wider comparisons show no statistical differences in incidence of postoperative complications. Indeed, postoperative complication rates within the closure group show great fluctuations, perhaps due to small sample sizes and differing study conditions. A rigorous, large sample study remains necessary to explore the effect of mesenteric closure on postoperative complications during LRH.

Initial inspection of data appeared to show a higher postoperative reoperation rate in patients retaining mesenteric defects than in those receiving closure. However, calculation of the confidence interval and construction of a forest plot indicate no statistically significant difference and the initially apparent difference may have been caused by sampling errors.

### Tools for mesenteric closure

Advantages and disadvantages of various tools were evaluated according to the authors’ experience (Table 3).

**Table 3:** Methods of mesenteric defects closure

Author	Year	Method	Advantage	Disadvantage	Into practice	Kinds of laparoscopic colorectal surgery mentioned in article
Cabot et al. [5]	2010	Using wound protector closure in an open fashion	Easy; time saving	May not produce safe and complete closure so may increase the risk of symptomatic internal herniation	Not in the article	Laparoscopic Right Colectomy
Sica et al. [14]	2021	Use of polymer ligating clips every 1–2 cm along the edges of the mesentery	Extremely resistant and easy to apply and remove	May result in adhesions due to foreign body reaction or migration	Yes	Laparoscopic Right Colectomy
Clapp et al. [13]	2020	Barbed suture	Time saving	Hard to remove; may cause intestinal tears or vascular injury	Yes	Laparoscopic Surgery
Anvari et al. [8]	2009	Non-absorbable suture or Ligasure™ device or staples	May lower incidence of internal hernias	In some particular situations using Ligasure™ device or staples may not be effective	Only non-absorbable sutures recommended	Laparoscopic Left Colectomy

Stefan [8], reported using both the Ligasure™ device and staples and also that use of a non-absorbable line achieved better results. All patients underwent laparoscopic left colon surgery. However one patient receiving closure via the Ligasure™ device experienced internal hernia due to incomplete closure and another patient who received staples had secondary rupture after closure. These two conditions are equally likely to apply to LRH, rendering the Ligasure™ device and staples less merit-worthy of further discussion. Complications related to barbed suture use, such as inflammation, were reported by Benjamin [13], indicating that careful consideration should accompany contemplated use of barbed sutures. Polymer-ligating clips were tested in 41 patients with no subsequent complications and thus are worthy of further consideration.

## Discussion

There is a paucity of clinical data relating to the prognosis of patients who have undergone LRH with mesenteric closure. No mesenteric defect-related complications were recorded for the current cohort of patients receiving closure but the possibility that some recorded complications were related to mesenteric defects cannot be excluded. 26 (4.9%) out of 530 patients retaining mesenteric defects experienced small bowel obstruction and 4 (0.8%) cases could be attributed to the mesenteric defects per se [5], There is undoubtedly less chance of small bowel obstruction after mesenteric closure in LRH. However, it is difficult to draw firm conclusions regarding the common occurrences of adhesion and bleeding in laparoscopic surgery [21]. Prognosis of laparoscopic right colon surgery is affected by surgical approach and method [22, 23]. Comparison of overall complications produces great statistical heterogeneity and rigorous large-scale randomized-control studies with attention paid to mechanisms are still required.

The data remains unclear as to whether closure makes a significant impact on complication and reoperation rates after LRH. In the three double-arm studies [15–17], there is a trend towards lower rates of re-operation following closure but the difference seems not to achieve statistical significance. Two of them have also conducted the patients with mesenteric defects are on the group that the conclusion does not support [16, 17]. Clinicians’ subjective views, confounding factors of the main research objectives and distortion or exaggeration of results due to sampling errors may all render conclusions unsafe. It is possible to arrive at a judgment in favor of closure, ignoring the absence of statistical differences between closure and non-closure for postoperative complications and reoperation. Such an observation provides a salutary reminder not to base opinions on the reading of a few impressive publications which, in the current case, might give the impression that mesenteric closure reduces the rate of reoperation.

The increasing scope of mesenteric resection seems to be a trend, making mesenteric closure more challenge. Even in LRH due to Crohn’s disease, mesenteric resection with laparoscopy has been widely adopted due to the promise it holds for reducing recurrence [24]. The wide use of Complete Mesocolic Excision(CME) in Asia, makes mesenteric closure harder in LRH. Mesenteric defects often result from surgery but may also be caused by weight loss and trauma [20]. Thus, a tool for easy suturing of mesenteric defects has great value. Tools referred to the literature include those which have not been proved in practice and carry some risk [5] and one which has been shown to have limitations in practice [8]. Barbed sutures may damage the intestinal tract and blood vessels and may not be superior to non-absorbable lines. Barbed sutures have been shown to be as effective as and to take a shorter time to use than non-absorbable wire in other operations [25, 26], but postoperative complications have also been reported [27]. Polymer ligation clip technology has been rarely used but its ease of use gives it a better fault tolerance rate. The current small sample size study attributes good results to polymer ligation clips and greater clinical attention is merited. Continuous suture has been shown superior to intermittent suture in bile duct surgery [28], and this may also be true for LRH. Anatomical characteristics of the mesentery mean that the small intestine is more inclined to the left iliac fossa. Maintaining this spatial arrangement during the perioperative period may render closure of the mesenteric defects unnecessary for LRH patients. There are theoretical advantages to closing the mesenteric defects and the slender small intestine of thin patients may easily pass through the mesenteric defect so that clinicians may opt for selective closure for thin patients [29]. Such observations allow clinicians to balance technical considerations and remove some obstacles through selective treatment. Mesenteric closure should be entertained by evaluation of overall complication rates and also by considering the individual patient’s condition.

Most prognostic considerations of LRH have focused on wound infection and intestinal obstruction [30, 31]. LRH patients differ from other laparoscopic colorectal surgery patients in that complications like wound infection and intestinal obstruction occur on a short post-operative time-scale. A study of thousands of cases concluded that internal hernia occurred several years after laparoscopic colorectal surgery, but observed an occurrence for 3 LRH patients at 3, 5 and 6 days post-operation [19]. Where appropriately strict regulations for regular post-operative outpatient review are in place, LRH complications due to mesenteric defects may be adequately monitored.

### Future of the field

Few studies on mesenteric closure in LRH have been conducted and more scrutiny of relevant risk confounders are required. The question of mesenteric closure in LRH remains and more work is needed to identify new tools or techniques for this purpose. A rigorously designed double arm observational study would fill the current gap.

### Limitations and strengths

The use of intra/extra-anastomosis and application of CME/D3 resection as influencing factors for LRH remain controversial [32–34]. Therefore, the current review focused on differences in postoperative complications and reoperation rates where stable influencing factors are difficult to identify. One of the two arm studies included in the current review had a small sample size and two other studies did not prioritize mesenteric closure. The remainder are single arm studies with a low level of evidence. We cannot exclude the possibility that we have missed some relevant literature but aimed to include all studies referring to mesenteric defects to offset the absence of quality assessment. However, the research field is young, meriting the current review to reveal existing data tendencies that may mislead clinicians and to collate surgical tool usage.

## Conclusion

No differences in postoperative complication or reoperation risks were found between patients receiving mesenteric closure and those retaining the mesenteric defect after LRH. Polymer-ligating clips for mesenteric closure in LRH are shown to shorten operation time, reduce operation difficulty and assist doctors in digestive tract reconstruction, but a randomized control trial remains necessary. Mesenteric closure cannot be recommended as a routine procedure due to the prolongation of operation time. Further large sample, real-world research remains necessary to make the current conclusions more authoritative.

## Data Availability

The datasets used and/or analyzed during the current study available from the corresponding author on reasonable request. The datasets we used for forest-plots can be seen in Tables 1 and 2 in this article. If you want more relevant files such as r code or something else, you can contact us.

## Abbreviations

LRH: Laparoscopic right hemicolectomy
